# Skin Needling to Enhance Depigmenting Serum Penetration in the Treatment of Melasma

**DOI:** 10.1155/2011/158241

**Published:** 2011-04-07

**Authors:** G. Fabbrocini, V. De Vita, N. Fardella, F. Pastore, M. C. Annunziata, M. C. Mauriello, A. Monfrecola, N. Cameli

**Affiliations:** ^1^Section of Dermatology, Department of Systematic Pathology, University of Naples Federico, Street Sergio Pansini 5, 80133 Napoli, Italy; ^2^San Gallicano Dermatological Institute, Street Elio Chianesi 53, 00144 Rome, Italy

## Abstract

Melasma is a common hypermelanotic disorder affecting the facial area which has a considerable psychological impact on the patient. Managing melasma is a difficult challenge that requires long-term treatment with a number of topical agents, such as rucinol and sophora-alpha. *Aims*. We aim to compare the combined treatment of skin needling and depigmenting serum with that using depigmenting serum alone in the treatment of melasma, in order to evaluate the use of microneedles as a means to enhance the drug's transdermal penetration. *Methods*. Twenty patients were treated with combined skin needling and depigmenting serum on one side of the face and with depigmenting serum alone on the other side. The outcome was evaluated periodically for up to two months using the Melasma Area Severity Index score and the Spectrocolorimeter X-Rite 968. *Results*. The side with combined treatment (skin needling + depigmenting serum) presented a statistically significant reduction in MASI score and luminosity index (L) levels compared to the side treated with depigmenting serum alone, and clinical symptoms were significantly improved. *Conclusions*. Our study suggests the potential use of combining skin needling with rucinol and sophora-alpha compounds to achieve better results in melasma treatment compared to rucinol and sophora-alpha alone.

## 1. Introduction

Melasma is an extremely common disorder in women between 20 and 45 years of age which involves alterations in normal skin pigmentation, resulting from the hyperactivity of epidermal melanocytes. It is exacerbated by sun exposure, pregnancy, oral contraceptives, and certain antiepilepsy drugs. The women most likely to develop melasma are those of fertile age with intermediate skin phototypes. Three histological pigmentation patterns have been identified: epidermal, in which the pigment is deposited in the basal or suprabasal layer; dermal, with melanin-laden macrophages in the superficial and middermis; and mixed, which is characterized by features of both the epidermal, and the dermal patterns. Hypermelanosis may be epidermal (brown), dermal (blue-gray), or mixed (brown-gray). Wood's lamp examination distinguishes epidermal from dermal hyperpigmentation in all skin phototypes except for V and VI, in which it is of no use. In skin phototypes I–IV, epidermal melasma is accentuated but dermal melasma is not. Clinically, the condition is characterized by irregularly shaped, asymptomatic spots ranging in color from beige to brown, which usually occur in the most photo-exposed areas: the upper lip, the cheeks, the cheekbones and the forehead. Its pathogenesis is not yet fully understood, but there is an association with genetic and hormonal factors, use of drugs and cosmetics, endocrinopathies, and sun exposure [1, 2].

The management of melasma is a challenge. Conventional melasma treatment includes elimination of any possible pathogenetic factors and the use of a sunscreen and hypopigmenting agent, often in combination with other therapies, such as tretinoin, topical corticosteroids, or superficial peeling agents. These treatments do not necessarily cure the cause of melasma, and the effectiveness of each will vary from patient to patient. Even after treatment, skin discoloration may not always disappear completely, and each patient may have to try various treatment options to achieve a satisfactory result. Some treatments may have to be performed continually in order to maintain results, such as regular application of a skin lightening agent combined with an effective use of sunscreen and avoidance of sun exposure.

Several lightening agents are available today, but hydroquinone, retinoic acid, and azelaic acid are the ones most frequently used. Combination therapies, for example, with hydroquinone, tretinoin, and corticosteroids, have also been used in the treatment of melasma and are thought to have greater efficacy than single therapies. Other investigations have been carried out on depigmenting agents' ability to block hypopigmentation or inhibit melanin synthesis through mechanisms involving tyrosinase synthesis inhibitors, tyrosinase activity inhibitors, cytotoxic agents for melanocytes, inhibitors of the transfer of melanosomes to keratinocytes, and so forth. New depigmenting products currently being used in cosmetics are rucinol and sophora-alpha. Rucinol inhibits the catalytic activity of tyrosinase and is also able to act on TRP-1 (tyrosinase-related protein 1) [3]; sophora-alpha, another lightening agent, acts on the outside of the melanocyte by blocking the extracellular action of alpha-MSH [4–6] (responsible for activating the melanin synthesis pathway by blocking MSH receptors). 

Er:YAG laser resurfacing, dermabrasion, and combined ultra-pulse CO_2_ laser with Q-switched alexandrite laser have been reported to show benefits for refractory melasma. However, these methods are more invasive, and posttreatment wound care is necessary. In addition, prolonged erythema, hyperpigmentation, hypopigmentation, infection, and hypertrophic scarring are potential side effects. Consequently, despite these measures, treatment of this recalcitrant disorder is often difficult and frustrating for the patient and the clinician.

In order to achieve better results in the treatment of melasma, in the last few years it has been proposed that the topical application of skin lightening agents should be combined with procedures to enhance the drugs' skin penetration, such as electroporation [7, 8], sonophoresis [9, 10], and iontophoresis [11, 12]. Recently, skin needling has been described as a new technique able to increase transdermal drug absorption [13–20]. 

The purpose of our study is to compare combined skin needling and depigmenting serum (containing two principal topical agents: rucinol and sophora-alpha) with depigmenting serum alone in the treatment of melasma in order evaluate the use of skin needling as a means to enhance the transdermal penetration of a serum containing rucinol and sophora-alpha in managing abnormal skin hyperpigmentation. 

## 2. Materials and Methods

### 2.1. Patients

Twenty female patients affected with melasma, involving both hemifaces, were recruited between September 2009 and October 2009. The patients were 32–60 years old (the average age was 53) and had Fitzpatrick skin types III–V, as shown in detail in Table 1. All patients signed the informed consent. The ethical committee approved the study (R.S. 36/09). Inclusion criteria were as follows: age between 32 and 60 years, bilateral and symmetrical idiopathic melasma, voluntary participation, ability to comprehend and provide informed consent, resistance to previous common therapies, preceding therapies discontinued at least 12 months before the start of the study, agreement not to use other therapies during the study. Exclusion criteria were as follows: history of keloid scarring, diabetes, bleeding disorder, collagen vascular disease, corticosteroid therapy, anticoagulant therapy, retionoid therapy, hormone therapy, presence of skin cancers, warts, solar keratoses, or any skin infection, current participation in another clinical study, any depigmenting treatment within the past 12 months, and lack of cooperation. Female volunteers were excluded if they were pregnant.

### 2.2. Procedure

During the first session (T0), each patient underwent a careful dermatologic examination: an experienced dermatologist clinically evaluated melasma areas on both hemifaces by using a Wood's lamp and the validated scoring method Melasma Area and Severity Index (MASI) [21]. The face of each patient was divided into four areas: forehead, right malar region, left malar region, and chin, corresponding to 30%, 30%, 30%, and 10% of the total face, respectively. The severity of the melasma in each of these four regions was assessed on the basis of three variables: percentage of the total area involved (A), darkness (D), and homogeneity (H). A numerical value was assigned for the corresponding percentage area involved: 0: no involvement; 1: <10% involvement; 2: 10–29% involvement; 3: 30–49% involvement; 4: 50–69% involvement; 5: 70–89% involvement; 6: 90–100% involvement. The darkness of the melasma (D) compared to normal skin and the homogeneity of the hyperpigmentation (H) were rated on a scale of 0 to 4 (0: normal skin color with no evidence of hyperpigmentation; 1: barely visible hyperpigmentation/specks of involvement; 2: mild hyperpigmentation/small patchy areas of involvement <1.5 cm diameter; 3: moderate hyperpigmentation/patches of involvement >2 cm diameter; 4: severe hyperpigmentation/uniform skin involvement without any clear areas). To calculate the MASI score, the sum of the severity grade for darkness (D) and homogeneity (H) was multiplied by the numerical value of the areas (A) involved and by the percentages of the four facial areas (10–30%). These values were summated to obtain the total MASI score: forehead 0.3 (D+H)A + right malar 0.3 (D+H)A + left malar 0.3 (D+H)A + Chin 0.1 (D+H)A.

**Figure 1:**
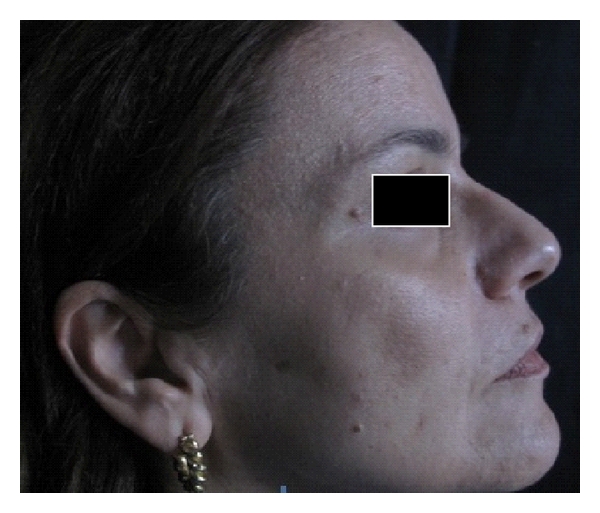
Digital standard photograph of a 44-year-old patient affected with melasma.

**Figure 2:**
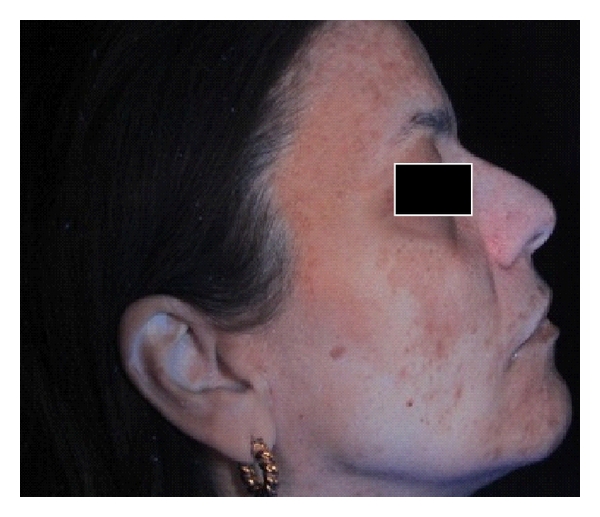
Digital UV photograph of a 44-year-old patient affected with melasma.

**Figure 3:**
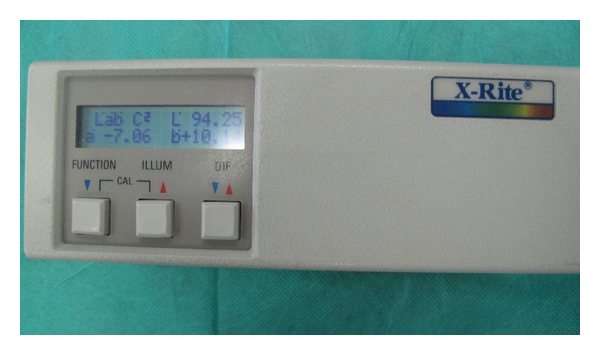
Spectrocolorimeter X-rite 968.

In order to carry out a comparative analysis, digital photographs were collected for each patient and gathered in a database: two right hemi-face and two left hemi-face photographs were captured by using both standard light and UV light; ultraviolet reflectance photography is a valuable tool to accentuate pigmentation (Figures 1 and 2), but patients must wash and degrease the face before photography to prevent reflection from the skin surface, which obscures pigmentation assessment. A colorimetric evaluation of melasma areas (right hemi-face, left hemi-face) was performed using X-Rite (Figure 3; Spectrocolorimeter X-rite 968). Colorimetric evaluation can objectively detect changes in the degree of skin pigmentation as the colorimeter records color in a designated three-dimensional space: L*, a*, b*. The first value, luminance (L*), expresses the brightness of the variations in color from total black to total white. The value a* is a tone of color ranging from red (+) to green (−). When the rash appears on the skin, the value becomes positive. The third value represented by b* is a tone of colour ranging from blue (−) to yellow (+). If hyperpigmentation appears, the value becomes positive. 

**Figure 4:**
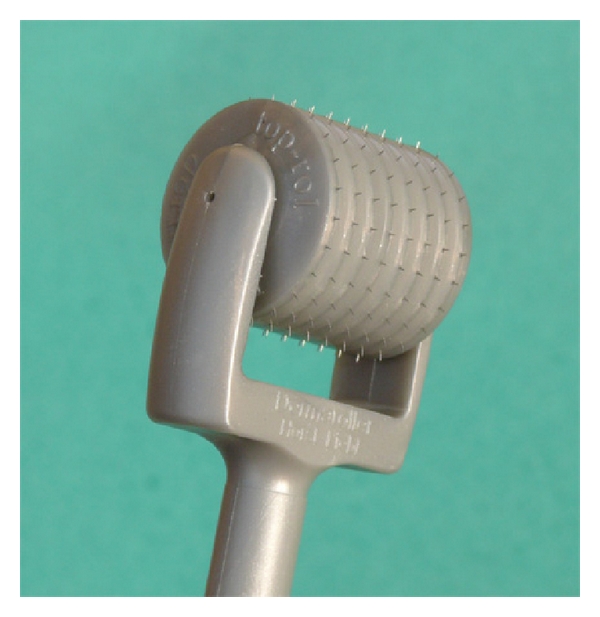
Dermaroller CIT 8: professional device.

The first treatment, performed by a different dermatologist, was preceded by the disinfection and the application of a topical anaesthetic for 60 minutes on melasma right hemifacial areas. A topical anaesthetic was also applied on melasma left hemi-facial areas for the same time period, so as not to introduce bias, as the PH of the topical anaesthetic should act as a factor against or favouring the penetration of the depigmenting agents used. This was carried out by rolling a special device over melasma areas, (Figure 4; Dermaroller-Model CIT 8) which consists of a 12 cm plastic handle attached to a cylinder, like a small paintroller, 20 mm in diameter and 20 mm in length. The surface of the cylinder houses 24 circular arrays of 8 needles each (total 192 needles), with a needle length of 0.5 mm and a diameter of 0.02 mm. Needles and disks are firmly bound together with a special medically approved adhesive. The tool was rolled in different directions: horizontally, vertically and diagonally, and right and left. This ensured an even pricking pattern, resulting in about 250–300 pricks/cm^2^. As expected, the skin bled for a short time after treatment. When bleeding stopped, a serous ooze formed and was removed from the surface of the skin using sterile saline solution. After rolling, the depigmenting serum was applied on the treated areas. On the melasma left hemi-facial areas, depigmenting serum alone was applied. This treatment was repeated twice with a one-month interval.

**Figure 5:**
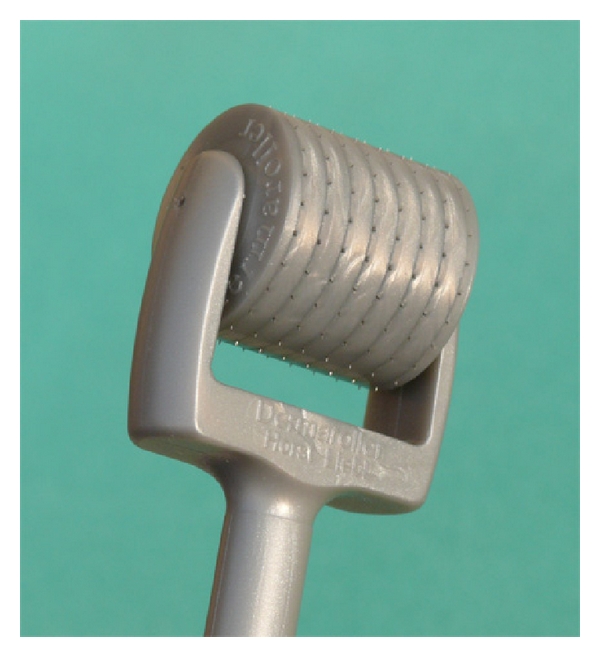
Dermaroller C8: home device.

In order to achieve better results, at the end of the first treatment, each patient was informed how to use the home roller device (Dermaroller-Model C8; Figure 5) and apply a depigmenting serum on melasma right hemi-facial areas. Model C8 consists of 196 needles arranged in 8 rows whose length is only 0.13 ± 0.02 mm, with the result that they blunt later than those in the device for medical use only so that the C8 model can be used at least 15 times without losing its skin penetration capacity. Use of C8 does not require local anaesthesia and does not cause bleeding. Patients performed this treatment every day for two months, rolling the home device horizontally, vertically and diagonally, and right and left, 8 times in each direction and immediately after applying a standardized amount of the depigmenting serum. The authors could approximately assess the patients' appropriate use of the device and the serum during this time by evaluating the time needed to blunt the microneedles and finish the serum. Furthermore, we recommended application of a total sunscreen on both hemi-faces. 

Each patient was examined one month after the first treatment: the same experienced dermatologist evaluated each patient's melasma areas on both hemi-faces, scoring them using the same scale as previously reported, to assess any clinical improvement in the severity of the lesions. Digital photographs and colorimetric evaluation of melasma areas were also collected and gathered in the database. The last followup visit was conducted in a similar manner two months after the first treatment. At this point, the patients' MASI score, digital photographs and colorimetric data were compared with the relative data collected during the first treatment.

## 3. Results

All patient completed the study. The results achieved after two sessions of treatment were assessed. After each treatment session, the facial skin treated using skin needling in combination with depigmenting serum appeared reddened and swollen, but patients stated that the redness and swelling disappeared in two to three days. No sideeffect was reported or found. All patients returned to work or normal activity immediately, and no patient needed to take time off work.

**Figure 6:**
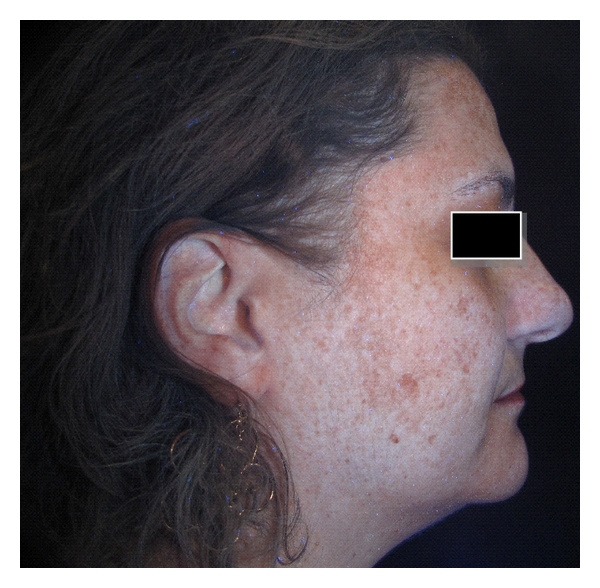
UV digital photograph of 42-year-old woman at baseline.

**Figure 7:**
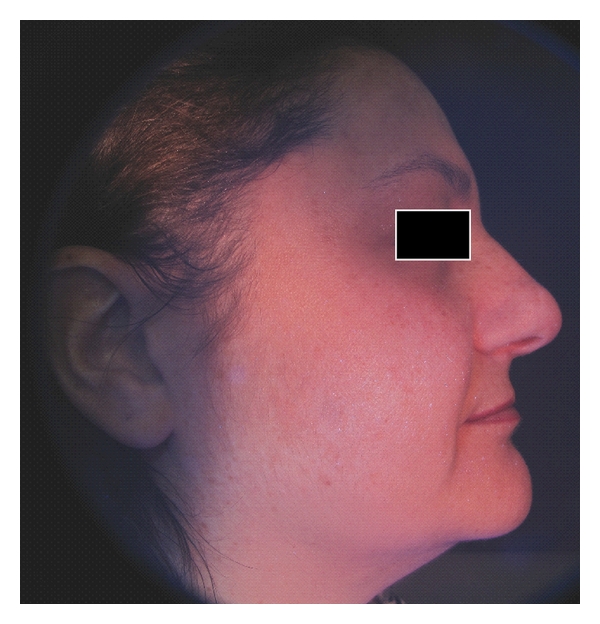
UV digital photograph of a 42-year-old woman treated by using skin needling with depigmenting serum (two months after the baseline).

**Figure 8:**
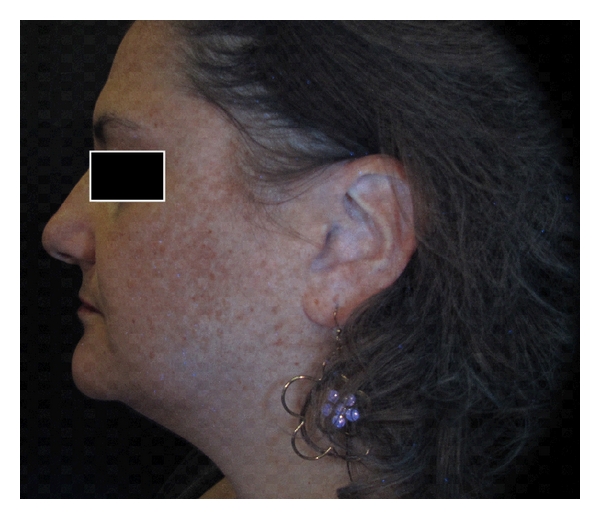
UV digital photograph of 42-year old woman at baseline.

**Figure 9:**
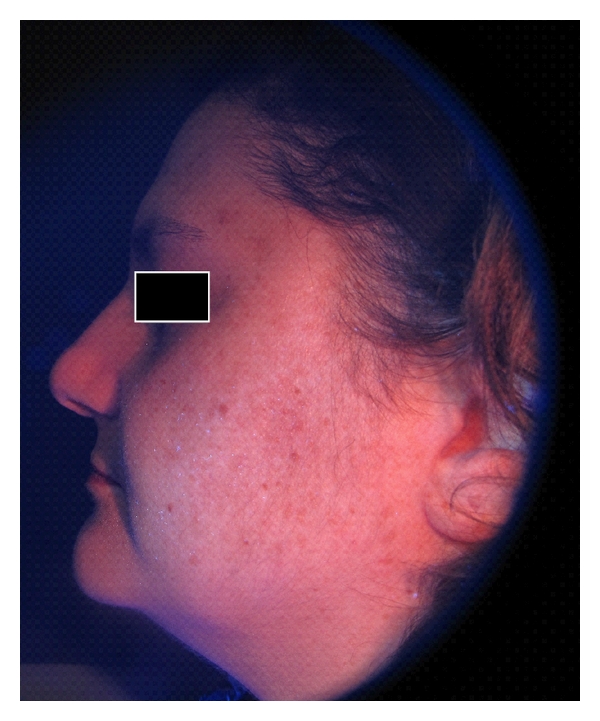
UV digital photograph of a 42-year-old woman treated by using depigmenting serum alone (two months after the baseline).

The photographic comparison highlighted that, in the areas treated with skin needling in combination with depigmenting serum, hyperpigmentation was significantly reduced compared to areas treated using depigmenting serum alone (Figures 6, 7, 8, and 9). On the right hemi-faces treated using the protocol therapy skin needling + depigmenting serum, the baseline mean MASI score of 19.1 decreased to 14.4 (*P* < .001) one month postoperatively and to 9.2 (*P* < .001) two months post operatively. On the left hemi-faces treated using depigmenting serum alone, the baseline mean MASI score of 20.4 decreased to 17.4 (*P* < .05) one month post operatively and to 13.3 two months post operatively (*P* < .05).

These results were confirmed by a statistically significant increase in the average luminance value (L*) in patients treated with skin needling and depigmenting serum (64.97) versus patients treated with depigmenting serum alone (61.61; paired Student's *t*-test; *P* < .05). Comparing luminance values (L*) before and after treatment, improvement translates into an increase in brightness of 17.4% in patients treated with skin needling and depigmenting serum; patients treated with depigmenting serum alone showed an increase in brightness of 11.2%, but it was less evident than the increase in brightness of the side treated with skin needling + depigmenting serum.

These results, shown in detail in Table 2, suggest that the use of skin needling improves the absorption of depigmenting serum and offers an important contribution in the treatment of melasma, which often constitutes a difficult challenge for dermatologists.

## 4. Discussion

Despite the existence of several depigmenting agents, the treatment of melasma is long and complicated, mainly because it is difficult for these substances to penetrate the skin. Indeed, the stratum corneum (SC), the outermost layer of the skin, is the main obstacle to the drugs' percutaneous absorption because its barrier function significantly restricts the drugs' transdermal delivery [22, 23]. It ranges from 10 to 20 *μ*m in thickness and consists of highly differentiated keratinocytes (corneocytes) embedded in an intercellular lipid matrix of mainly fatty acids, ceramides, cholesterol, and cholesterol sulfate. To penetrate into the skin, drug molecules must be small in size and/or low molecular weight. Lipophilic molecules can penetrate the skin deeper than hydrophilic ones. In the last few years, the transdermal delivery of active substances has become an important therapy used in treating a large number of skin diseases. 

Skin penetration enhancement can be achieved either physically or chemically [24–27]. Many techniques have been developed to improve transdermal drug delivery, such as electroporation [7, 8], sonophoresis [9, 10], and iontophoresis [11, 12], which are able to improve the stratum corneum layer permeability and to enhance penetration of topical agents through the skin.

Recently, the use of skin needling has been proposed as a new physical strategy to increase transdermal drug delivery. Since, 1995 this technique has been used to achieve percutaneous collagen induction in order to reduce skin imperfections [28, 29]. To date, skin needling has mostly been proposed as an effective method of treating scars and wrinkles [30, 31], and it is carried out by rolling a special device over the skin comprising a rolling barrel fitted with a variable number of microneedles. The micro-needles penetrate through the epidermis but do not remove it; the epidermis is only punctured and heals rapidly. The needles seem to separate the cells from one another rather than cut through them, and thus many cells are spared. Because the needles are set in a roller, every needle initially penetrates at an angle and then goes deeper as the roller turns. Finally, the needle is extracted at a converse angle, therefore curving the tracts and reflecting the path of the needle as it rolls into and then out of the skin for about 0.5 mm into the dermis. The epidermis, and particularly the stratum corneum, remains intact except for the minute holes, which are about four cells in diameter [32]. In 1998, Henry et al., in the first study on microneedle use for transdermal drug delivery, showed an increase in magnitude of four levels in the permeability of human skin after the insertion of an array of 150 *μ*m long, solid silicon, out-of-plane microneedles [33]. More recently, several authors have shown that microneedle-injected sites have a significantly higher transdermal penetration [13–20]. Recent reports have tried to identify the mechanisms involved in the enhancement of transdermal drug delivery and several hypotheses have been proposed, though none is completely exhaustive. 

To understand the mechanism by which microneedles increase skin permeability, McAllister et al. theoretically modeled transdermal transport as diffusion through holes of known geometry made by insertion of microneedles. All the scientific data is based on a repetitive rolling (10 to 15 times) on the same area of the skin. In this case around 240 microinfiltration pores per square centimeter are set and all of these pores close within minutes [32]. 

It may be that the microneedles aid in bypassing the stratum corneum and enhancing drug delivery through the skin by increasing skin blood perfusion also. A Laser Doppler Perfusion Monitor was used to record maximum blood flow and the time needed to reach maximum blood flow in the treatment areas. Sections treated with microneedles showed a higher maximum blood flow and reached maximum blood flow faster than sites not treated with microneedles [19].

## 5. Conclusions

This pilot study describes the first report of improvement in melasma through the use of skin needling in combination with a depigmenting serum, and shows that combination therapy with skin needling and topical depigmenting serum is more effective than topical depigmenting serum alone in improving melasma. As this was a pilot study, we have neither a large sample size nor a long-term followup; a larger sample and longer followups are needed to assess the long-term efficacy of our results. Potential refinement of the number of sessions and treatment parameters call for further evaluation in order to maximize the therapeutic efficacy of this new way of treating melasma, but it opens new perspectives for employment of this device to enhance the penetration of depigmenting compounds and to reduce treatment times.

## Figures and Tables

**Table 1:** 

	No. of patients (%)
Age (years)	
32–40	2 (10)
41–50	8 (40)
51–60	10 (50)
Fitzpatrick skin type	
III	6 (30)
IV	9 (45)
V	5 (25)

**Table 2:** 

	Depigmenting Serum + Skin needling		Depigmenting serum alone	
Pz.	L* before treatment	L* after treatment	L* before treatment	L* after treatment

1	51.70	59.89	54.80	59.94
2	55.82	65.49	55.36	60.88
3	58.45	68.22	56.77	64.89
4	54.27	64.17	56.67	63.74
5	48.85	59.63	53.15	59.55
6	59.78	70.92	61.18	62.18
7	55.09	66.30	53.92	60.97
8	52.10	63.70	51.20	57.66
9	56.73	69.27	58.18	65.52
10	56.34	67.18	59.48	65.02
11	51.45	62.02	54.54	60.55
12	60.15	70.38	59.76	66.61
13	52.60	63.70	54.39	60.03
14	56.25	64.22	56.10	59.00
15	55.91	66.10	56.83	62.03
16	50.26	65.35	55.92	59.62
17	52.73	59.73	52.13	60.79
18	50.35	64.71	51.17	59.22
19	52.72	62.71	54.73	62.14
20	55.31	65.73	52.04	61.98
Average	54.34	64.97	55.42	61.61
D STD	3.2665	3.1710	2.8177	2.425
